# Supplementary material for: Contrasting molecular and morphological evidence for the identification of an anomalous Buteo: a cautionary tale for hybrid diagnosis
Source: PeerJ. 2017 Jan 10;5:e2850. doi: 10.7717/peerj.2850 (PMC5228515; doi:10.7717/peerj.2850)
Supplement: Appendix S1 [file peerj-05-2850-s001.docx]

**Appendix 1.** Morphometric data used in the PCA. Museum acronyms in specimen identifiers include AMNH (American Museum of Natural History), MSB (Museum of Southwestern Biology of the University of New Mexico), WFVZ (Western Foundation of Vertebrate Zoology), and WFB (Museum of Wildlife and Fish Biology of the University of California-Davis).

| Specimen | Taxon | Sex | Wing chord (mm) | Culmen (mm) | Tarsus (mm) | Hallux (mm) | Tail (mm) | State or Province |
| --- | --- | --- | --- | --- | --- | --- | --- | --- |
| WFVZ:Bird:20607 | *B. j. calurus* | f | 408 | 27 | 89.5 | 27.4 | 206 | CA |
| WFVZ:Bird:32972 | *B. j. calurus* | f | 419 | 27.5 | 97.5 | 30.4 | 229 | CA |
| WFVZ:Bird:36209 | *B. j. calurus* | f | 400 | 26.9 | 99.6 | 29.7 | 217 | CA |
| WFVZ:Bird:39 | *B. j. calurus* | f | 394 | 25.7 | 90.9 | 28.9 | 221 | CA |
| WFVZ:Bird:53726 | *B. j. calurus* | f | 407 | 27.8 | 96.2 | 28.8 | 222 | CA |
| WFVZ:Bird:53990 | *B. j. calurus* | f | 419 | 23.6 | 101.5 | 27.7 | 225 | CA |
| WFVZ:Bird:54010 | *B. j. calurus* | f | 428 | 24.5 | 108.6 | 29.8 | 230 | CA |
| WFVZ:Bird:57218 | *B. j. calurus* | f | 403 | 25.7 | 98.5 | 28.4 | 220 | CA |
| WFVZ:Bird:5846 | *B. j. calurus* | f | 404 | 26.5 | 90.2 | 27.5 | 217 | CA |
| WFVZ:Bird:6000 | *B. j. calurus* | f | 419 | 26.3 | 90.5 | 29.4 | 221 | CA |
| WFVZ:Bird:6337 | *B. j. calurus* | f | 401 | 24.2 | 96.8 | 28.5 | 223 | CA |
| WFVZ:Bird:705 | *B. j. calurus* | f | 417 | 26.7 | 91.4 | 30.2 | 218 | CA |
| WFVZ:Bird:20579 | *B. j. calurus* | m | 377 | 23.6 | 95.5 | 28.3 | 215 | CA |
| WFVZ:Bird:32565 | *B. j. calurus* | m | 387 | 24.2 | 95.8 | 27.6 | 203 | CA |
| WFVZ:Bird:32936 | *B. j. calurus* | m | 391 | 24.2 | 95.2 | 27.2 | 217 | CA |
| WFVZ:Bird:37106 | *B. j. calurus* | m | 365 | 25.5 | 92.8 | 26.6 | 199 | CA |
| WFVZ:Bird:43362 | *B. j. calurus* | m | 385 | 24.9 | 90.6 | 27.4 | 202 | CA |
| WFVZ:Bird:45305 | *B. j. calurus* | m | 396 | 24.2 | 91.8 | 27.3 | 198 | CA |
| WFVZ:Bird:53764 | *B. j. calurus* | m | 397 | 26.3 | 95.3 | 29.8 | 196 | CA |
| WFVZ:Bird:56775 | *B. j. calurus* | m | 373 | 24.2 | 93.3 | 28.8 | 194 | CA |
| WFVZ:Bird:6517 | *B. j. calurus* | m | 369 | 23.3 | 90.3 | 27.3 | 204 | CA |
| WFVZ:Bird:6642 | *B. j. calurus* | m | 394 | 24.3 | 95.1 | 27.4 | 212 | CA |
| WFVZ:Bird:6711 | *B. j. calurus* | m | 385 | 23.9 | 94.3 | 28.3 | 202 | CA |
| WFVZ:Bird:154 | *B. l. elegans* | f | 317 | 20.4 | 84.2 | 22.8 | 193 | CA |
| WFVZ:Bird:31 | *B. l. elegans* | f | 314 | 20.9 | 81 | 22.7 | 199 | CA |
| WFVZ:Bird:44650 | *B. l. elegans* | f | 307 | 21.2 | 84 | 20.6 | 191 | CA |
| WFVZ:Bird:44651 | *B. l. elegans* | f | 320 | 22.4 | 86.1 | 26.2 | 194 | CA |
| WFVZ:Bird:52711 | *B. l. elegans* | f | 318 | 21 | 84.2 | 22.7 | 189 | CA |
| WFVZ:Bird:53466 | *B. l. elegans* | f | 307 | 20.7 | 85.8 | 20.7 | 193 | CA |
| WFVZ:Bird:53525 | *B. l. elegans* | f | 296 | 21.7 | 84.3 | 22.3 | 190 | CA |
| WFVZ:Bird:53751 | *B. l. elegans* | f | 289 | 20.1 | 77.5 | 20.5 | 182 | CA |
| WFVZ:Bird:53752 | *B. l. elegans* | f | 310 | 20.7 | 81.6 | 22.6 | 192 | CA |
| WFVZ:Bird:54013 | *B. l. elegans* | f | 321 | 21.3 | 85.4 | 21.7 | 209 | CA |
| WFVZ:Bird:56778 | *B. l. elegans* | f | 300 | 22.2 | 86.8 | 22.5 | 182 | CA |
| WFVZ:Bird:56964 | *B. l. elegans* | f | 311 | 20 | 79 | 21.4 | 192 | CA |
| WFVZ:Bird:5985 | *B. l. elegans* | f | 312 | 23.2 | 82 | 23 | 202 | CA |
| WFVZ:Bird:6156 | *B. l. elegans* | f | 314 | 21.3 | 80.7 | 22.7 | 196 | CA |
| WFVZ:Bird:6274 | *B. l. elegans* | f | 310 | 25 | 85.4 | 23 | 203 | CA |
| WFVZ:Bird:710 | *B. l. elegans* | f | 301 | 22.6 | 82.3 | 22.2 | 186 | CA |
| MSB:Bird:25344 | *B. l. elegans* | m | 293 | 19.56 | 78.53 | 21.17 | 208 | CA |
| WFVZ:Bird:29384 | *B. l. elegans* | m | 291 | 20.2 | 78.6 | 22.8 | 180 | CA |
| WFVZ:Bird:32971 | *B. l. elegans* | m | 300 | 19.7 | 85 | 18.2 | 173 | CA |
| WFVZ:Bird:3949 | *B. l. elegans* | m | 302 | 20.3 | 84.8 | 23.1 | 191 | CA |
| WFVZ:Bird:42145 | *B. l. elegans* | m | 283 | 20 | 82.6 | 19.9 | 172 | CA |
| WFVZ:Bird:43374 | *B. l. elegans* | m | 297 | 17.8 | 77.2 | 19 | 176 | CA |
| WFVZ:Bird:45273 | *B. l. elegans* | m | 278 | 20.8 | 82.3 | 20.2 | 177 | CA |
| WFVZ:Bird:5038 | *B. l. elegans* | m | 292 | 20.6 | 78.5 | 18.8 | 180 | CA |
| WFVZ:Bird:52712 | *B. l. elegans* | m | 296 | 19.2 | 79.7 | 21.5 | 181 | CA |
| WFVZ:Bird:53484 | *B. l. elegans* | m | 292 | 18.2 | 76.4 | 17.8 | 173 | CA |
| WFVZ:Bird:56777 | *B. l. elegans* | m | 289 | 20.5 | 80.5 | 20.2 | 187 | CA |
| WFVZ:Bird:5835 | *B. l. elegans* | m | 287 | 19 | 76.5 | 19.2 | 173 | CA |
| WFVZ:Bird:5845 | *B. l. elegans* | m | 292 | 19.4 | 81.1 | 20.3 | 172 | CA |
| WFVZ:Bird:5921 | *B. l. elegans* | m | 286 | 19.5 | 77.3 | 21.3 | 184 | CA |
| WFVZ:Bird:5933 | *B. l. elegans* | m | 278 | 20.8 | 83 | 21.4 | 168 | CA |
| WFVZ:Bird:6241 | *B. l. elegans* | m | 302 | 18.9 | 81.5 | 20.1 | 181 | CA |
| WFVZ:Bird:6519 | *B. l. elegans* | m | 280 | 18.5 | 78.7 | 20.2 | 185 | CA |
| AMNH:Bird:168613 | *B. lineatus* (eastern) | f | 352 | 24.42 | 80.85 | 22.92 | 211 | NY |
| AMNH:Bird:352521 | *B. lineatus* (eastern) | f | 354 | 24.44 | 79.23 | 23.86 | 204.13 | IA |
| AMNH:Bird:352553 | *B. lineatus* (eastern) | f | 355 | 23.95 | 81.13 | 23.18 | 208.8 | NH |
| AMNH:Bird:352559 | *B. lineatus* (eastern) | f | 347 | 22.71 | 80.8 | 22.81 | 199.56 | ON |
| AMNH:Bird:470684 | *B. lineatus* (eastern) | f | 358 | 22.94 | 80.46 | 21.45 | 208.6 | ON |
| AMNH:Bird:69780 | *B. lineatus* (eastern) | f | 359 | 20.24 | 85.4 | 23.9 | 198.45 | NJ |
| AMNH:Bird:69781 | *B. lineatus* (eastern) | f | 346 | 22.06 | 81.04 | 22.25 | 213 | MD |
| AMNH:Bird:750225 | *B. lineatus* (eastern) | f | 355 | 21.31 | 75.5 | 21.6 | 208.8 | CT |
| AMNH:Bird:832602 | *B. lineatus* (eastern) | f | 359 | 23.4 | 84.3 | 23.25 | 211 | RI |
| AMNH:Bird:96626 | *B. lineatus* (eastern) | f | 328 | 20 | 74.66 | 21.66 | 200.67 | NY |
| MSB:Bird:24325 | *B. lineatus* (eastern) | f | 321 | 21.5 | 74.45 | 20.89 | 194 | LA |
| MSB:Bird:24885 | *B. lineatus* (eastern) | f | 307 | 22.22 | 77.14 | 21.81 | 185 | FL |
| MSB:Bird:4291 | *B. lineatus* (eastern) | f | 352 | 22.55 | 76.81 | 22.78 | 211 | IA |
| WFVZ:Bird:20446 | *B. lineatus* (eastern) | f | 340 | 21.5 | 84.9 | 21.7 | 200 | PA |
| WFVZ:Bird:20447 | *B. lineatus* (eastern) | f | 350 | 21.1 | 82.5 | 21.8 | 203 | GA |
| WFVZ:Bird:20464 | *B. lineatus* (eastern) | f | 335 | 22.2 | 84.6 | 23.1 | 195 | GA |
| WFVZ:Bird:20472 | *B. lineatus* (eastern) | f | 325 | 20.7 | 84.7 | 19.5 | 207 | NJ |
| WFVZ:Bird:20476 | *B. lineatus* (eastern) | f | 345 | 21.9 | 84 | 21.5 | 209 | MD |
| WFVZ:Bird:20477 | *B. lineatus* (eastern) | f | 341 | 20.1 | 86.8 | 20.7 | 213 | NJ |
| WFVZ:Bird:20481 | *B. lineatus* (eastern) | f | 343 | 20.6 | 84.9 | 22.3 | 205 | PA |
| WFVZ:Bird:20483 | *B. lineatus* (eastern) | f | 327 | 19.7 | 81.7 | 21.1 | 204 | PA |
| WFVZ:Bird:20490 | *B. lineatus* (eastern) | f | 337 | 21.3 | 84.8 | 19.2 | 181 | FL |
| WFVZ:Bird:47830 | *B. lineatus* (eastern) | f | 350 | 22.9 | 81.9 | 22.7 | 214 | NC |
| WFVZ:Bird:49754 | *B. lineatus* (eastern) | f | 324 | 20 | 84.2 | 22.2 | 197 | TX |
| AMNH:Bird:234136 | *B. lineatus* (eastern) | m | 316 | 20.22 | 75.81 | 20.43 | 188.47 | ON |
| AMNH:Bird:352520 | *B. lineatus* (eastern) | m | 339 | 20.24 | 80.71 | 20.79 | 200.19 | IA |
| AMNH:Bird:352526 | *B. lineatus* (eastern) | m | 346 | 21.14 | 82.17 | 20.11 | 194.75 | MO |
| AMNH:Bird:352531 | *B. lineatus* (eastern) | m | 335 | 22.45 | 77.9 | 20.72 | 195.57 | NJ |
| AMNH:Bird:352535 | *B. lineatus* (eastern) | m | 333 | 22.82 | 79.21 | 21.27 | 184.32 | NY |
| AMNH:Bird:470683 | *B. lineatus* (eastern) | m | 330 | 19.69 | 73.18 | 20.41 | 202.38 | ON |
| AMNH:Bird:832603 | *B. lineatus* (eastern) | m | 324 | 21.74 | 79.52 | 21.11 | 193.77 | MA |
| AMNH:Bird:832604 | *B. lineatus* (eastern) | m | 337 | 21.71 | 80.14 | 20.91 | 200.7 | RI |
| AMNH:Bird:836423 | *B. lineatus* (eastern) | m | 341 | 19.91 | 74 | 20.38 | 195.24 | RI |
| AMNH:Bird:98112 | *B. lineatus* (eastern) | m | 334 | 21.11 | 77.29 | 21.67 | 197 | MA |
| MSB:Bird:21684 | *B. lineatus* (eastern) | m | 306 | 19.57 | 73.52 | 21.63 | 207 | TX |
| WFVZ:Bird:20461 | *B. lineatus* (eastern) | m | 321 | 19.5 | 83.1 | 22.2 | 194 | PA |
| WFVZ:Bird:20465 | *B. lineatus* (eastern) | m | 329 | 19.1 | 79.2 | 21 | 183 | NJ |
| WFVZ:Bird:20470 | *B. lineatus* (eastern) | m | 310 | 20 | 79.8 | 19.8 | 185 | GA |
| WFVZ:Bird:20471 | *B. lineatus* (eastern) | m | 319 | 20.8 | 77 | 18.4 | 189 | GA |
| WFVZ:Bird:20472 | *B. lineatus* (eastern) | m | 308 | 18.8 | 78.5 | 20.3 | 184 | PA |
| WFVZ:Bird:20473 | *B. lineatus* (eastern) | m | 323 | 21.2 | 84.8 | 19.9 | 195 | GA |
| WFVZ:Bird:20474 | *B. lineatus* (eastern) | m | 313 | 19.5 | 79 | 21.9 | 186 | GA |
| WFVZ:Bird:20497 | *B. lineatus* (eastern) | m | 311 | 20 | 75 | 20 | 192 | GA |
| WFVZ:Bird:20499 | *B. lineatus* (eastern) | m | 315 | 19.7 | 75.9 | 19.7 | 185 | GA |
| WFVZ:Bird:48010 | *B. lineatus* (eastern) | m | 302 | 18.9 | 78.3 | 18.4 | 186 | GA |
| WFVZ:Bird:50191 | *B. lineatus* (eastern) | m | 318 | 20.2 | 76.7 | 21.7 | 197 | TX |
| WFB:Bird:4816 | Putative hybrid | n/a | 350 | 23.9 | 83.2 | 25.12 | 212 | CA |
