# Supplementary material for: Contrasting molecular and morphological evidence for the identification of an anomalous Buteo: a cautionary tale for hybrid diagnosis
Source: PeerJ. 2017 Jan 10;5:e2850. doi: 10.7717/peerj.2850 (PMC5228515; doi:10.7717/peerj.2850)
Supplement: Appendix S2 [file peerj-05-2850-s002.docx]

**Appendix 2.** Measurements of hallux chord from 98 adult female *B. l. lineatus* specimens (x̄= 22.1, SD = 1.0, range 19.2-23.9). Museum acronyms in specimen identifiers include AMNH (American Museum of Natural History), BMUW (University of Washington Burke Museum), MSB (Museum of Southwestern Biology of the University of New Mexico), MVZ (Museum of Vertebrate Zoology of the University of California-Berkeley), USNM (Smithsonian Institution), WFVZ (Western Foundation of Vertebrate Zoology), and WFB (Museum of Wildlife and Fish Biology of the University of California-Davis).

| Specimen | Hallux (mm) | State/Province/Country |
| --- | --- | --- |
| USNM:Bird:41683 | 22 | DC |
| USNM:Bird:1278 | 22.9 | PA |
| USNM:Bird:372246 | 20.7 | LA |
| USNM:Bird:74360 | 21.8 | Mexico |
| USNM:Bird:84511 | 21.4 | IL |
| USNM:Bird:84512 | 21.3 | IL |
| USNM:Bird:85456 | 23.9 | IL |
| USNM:Bird:101620 | 23.3 | PA |
| USNM:Bird:121489 | 23.2 | PA |
| USNM:Bird:124977 | 22.6 | MD |
| USNM:Bird:141121 | 22.2 | MD |
| USNM:Bird:160852 | 23.7 | GA |
| USNM:Bird:176027 | 22 | MD |
| USNM:Bird:204909 | 23.9 | MD |
| USNM:Bird:231534 | 22.2 | IL |
| USNM:Bird:257191 | 22.9 | AR |
| USNM:Bird:271233 | 21.3 | MD |
| USNM:Bird:271235 | 22.7 | NJ |
| USNM:Bird:272298 | 22.7 | NJ |
| USNM:Bird:293611 | 21.9 | MD |
| USNM:Bird:293613 | 20.9 | MD |
| USNM:Bird:302987 | 23.6 | NC |
| USNM:Bird:305541 | 21.2 | MD |
| USNM:Bird:307940 | 22.8 | MD |
| USNM:Bird:309401 | 21.6 | VA |
| USNM:Bird:309404 | 21.7 | NJ |
| USNM:Bird:311751 | 20.6 | NJ |
| USNM:Bird:313564 | 20.6 | PA |
| USNM:Bird:333275 | 20.9 | VA |
| USNM:Bird:337332 | 23.4 | KY |
| USNM:Bird:350854 | 22.5 | TN |
| USNM:Bird:350855 | 22.1 | TN |
| USNM:Bird:358210 | 22.6 | TN |
| USNM:Bird:361787 | 21.8 | NC |
| USNM:Bird:361788 | 21.7 | SC |
| USNM:Bird:361790 | 22.2 | SC |
| USNM:Bird:363767 | 20.4 | SC |
| USNM:Bird:378649 | 23.6 | GA |
| USNM:Bird:378763 | 21.6 | MD |
| USNM:Bird:414277 | 20.6 | NY |
| USNM:Bird:419685 | 22.5 | NY |
| USNM:Bird:479274 | 21.7 | OK |
| USNM:Bird:524102 | 21.7 | IA |
| USNM:Bird:524104 | 23.4 | MD |
| USNM:Bird:524105 | 20.5 | MD |
| USNM:Bird:524107 | 22.5 | MD |
| USNM:Bird:524108 | 22.1 | MD |
| USNM:Bird:524111 | 21.3 | MD |
| USNM:Bird:532317 | 22.9 | MD |
| USNM:Bird:565400 | 21.9 | IN |
| USNM:Bird:567311 | 21.8 | IN |
| USNM:Bird:588436 | 22.2 | PA |
| USNM:Bird:599472 | 22.2 | MD |
| USNM:Bird:602033 | 22.9 | VA |
| BMUW:Bird:79527 | 20.1 | VA |
| BMUW:Bird:76102 | 23.2 | NC |
| FMNH:Bird:300768 | 19.9 | MD |
| FMNH:Bird:130444 | 21.1 | NJ |
| FMNH:Bird:130416 | 21.5 | GA |
| FMNH:Bird:67098 | 21.7 | CT |
| FMNH:Bird:324665 | 21.8 | IL |
| FMNH:Bird:300779 | 21.9 | IL |
| FMNH:Bird:435708 | 22.2 | NJ |
| FMNH:Bird:16203 | 22.3 | IL |
| FMNH:Bird:130422 | 22.4 | CT |
| FMNH:Bird:74686 | 22.5 | CT |
| FMNH:Bird:88367 | 23 | AR |
| FMNH:Bird:20914 | 23.5 | IL |
| MVZ:Bird:99787 | 20.7 | CT |
| MVZ:Bird:144675 | 20.8 | IL |
| MVZ:Bird:58416 | 21.5 | SC |
| MVZ:Bird:127228 | 22.2 | KS |
| MVZ:Bird:127231 | 23.4 | MA |
| MVZ:Bird:94366 | 23.7 | MA |
| AMNH:Bird:168613 | 22.9 | NY |
| AMNH:Bird:352251 | 23.9 | IA |
| AMNH:Bird:352553 | 23.2 | NH |
| AMNH:Bird:352559 | 22.8 | ON |
| AMNH:Bird:470684 | 21.5 | ON |
| AMNH:Bird:69780 | 23.9 | NJ |
| AMNH:Bird:69781 | 22.3 | MD |
| AMNH:Bird:750225 | 21.6 | CT |
| AMNH:Bird:832602 | 23.3 | RI |
| AMNH:Bird:96626 | 21.7 | NY |
| MSB:Bird:24325 | 20.9 | LA |
| MSB:Bird:24885 | 21.8 | FL |
| MSB:Bird:4291 | 22.8 | LA |
| WFVZ:Bird:20446 | 21.7 | PA |
| WFVZ:Bird:20447 | 21.8 | GA |
| WFVZ:Bird:20464 | 23.1 | GA |
| WFVZ:Bird:20472 | 19.5 | NJ |
| WFVZ:Bird:20476 | 21.5 | MD |
| WFVZ:Bird:20477 | 20.7 | NJ |
| WFVZ:Bird:20481 | 22.3 | PA |
| WFVZ:Bird:20483 | 21.1 | PA |
| WFVZ:Bird:20490 | 19.2 | FL |
| WFVZ:Bird:47830 | 22.7 | NC |
| WFVZ:Bird:47954 | 22.2 | TX |
