# Supplementary material for: Contrasting molecular and morphological evidence for the identification of an anomalous Buteo: a cautionary tale for hybrid diagnosis
Source: PeerJ. 2017 Jan 10;5:e2850. doi: 10.7717/peerj.2850 (PMC5228515; doi:10.7717/peerj.2850)
Supplement: Appendix S3 [file peerj-05-2850-s003.docx]

Appendix ##. ND2 sequence and GenBank accession number obtained from voucher specimen WFB 4816.

| **Voucher No.** | **Species** | **ND2 Sequence** | **GenBank Accession No.** |
| --- | --- | --- | --- |
| WFB 4816 | *Buteo lineatus* | TCTGAACAAAATCCCCCACACTAAACGCAACCCTCATACTAGTCCTACTTTCCCTAGCAGGCCTCCCACCTCTAACAGGCTTCCTACCCAAATGATTGATTATCCAAGAACTAACCAAACAAGAAATAGCCACAACAGCTACAATCCTCGCTACCCTCTCCCTCCTGGGGTTATTCTTCTACCTCCGCCTCGA | KX154215 |
